# The Role of Repeated Surgical Resections for Recurrent Brain Metastases in Older Population

**DOI:** 10.3390/medicina60091464

**Published:** 2024-09-06

**Authors:** Maria Goldberg, Valeri Heinrich, Ghaith Altawalbeh, Chiara Negwer, Arthur Wagner, Jens Gempt, Bernhard Meyer, Amir Kaywan Aftahy

**Affiliations:** 1 Department of Neurosurgery, School of Medicine, Klinikum Rechts der Isar, Technical University Munich, 81675 Munich, Germany; 2 Department of Neurosurgery, University Medical Center Hamburg-Eppendorf, 20246 Hamburg, Germany; j.gempt@uke.de

**Keywords:** brain metastases, neurosurgical resection, older patients, recurrent brain metastases

## Abstract

*Background and Objectives:* The impact of surgery for recurrent brain metastases in elderly patients has been the object of debate due to limited information in the literature. We analyzed clinical outcome and survival of elderly patients with recurrent brain metastases in order to assess potentially beneficial role of surgery. *Materials and methods:* In total, 219 patients with recurrent brain metastases between 2007 and 2022 were identified, of which 95 underwent re-resection; 83 patients aged 65 and older were analyzed. A survival analysis was performed, and clinical outcomes were evaluated. *Results:* The median survival time after surgery for recurrent brain metastases was 6 months (95CI 4–10) in older patients and 8 (95CI 7–9) in younger patients (*p* = 0.619). Out of all the older patients, 33 who underwent surgical resection showed prolonged survival compared with patients who did not receive surgical resection (median: 14, 95CI 8–19 vs. 4, 95CI 4–7, *p* = 0.011). All patients had preoperative Karnofsky performance scores of >70, which did not deteriorate after surgery (87.02 ± 5.76 vs. 85 ± 6.85; *p* = 0.055). In the univariate analysis, complete cytoreduction was a favorable prognostic factor. The tumor volume, the number of metastases, extracranial disease progression, adjuvant radiation, and systemic therapy did not affect survival in this cohort. *Conclusions:* Patients aged 65 and older benefit from neurosurgical resections of recurrent brain metastases. Survival did not differ from that in younger patients, which can be explained by a better preoperative functional status. Moreover, independent of the extent of resection, older patients who underwent surgery showed better survival than patients who did not receive surgical treatment. Complete cytoreduction was a favorable prognostic marker.

## 1. Introduction

Brain metastases occur in nearly one-third of cancer patients [1], and the incidence of brain tumors in general increases in elderly patients [2]. The most frequently diagnosed age group for synchronous brain metastasis is 60 to 69 years (33%), followed by 70 to 79 years (24.5%) and over 80 years (11.2%), making the older patient population the most represented group [3]. The rising interest in elderly patients has prompted numerous studies, revealing that advanced age (≥65 years) alone is not an independent risk factor for worse outcomes [4]. The extended survival of the aging population, along with breakthroughs in systemic therapy for older patients, necessitates a re-evaluation of surgical outcomes in brain metastases [5]. Brain metastases pose a significant challenge in the treatment of elderly cancer patients, primarily due to the higher incidence of these tumors in this population group and the complex interplay of comorbidities and age-related vulnerabilities. The inclination towards surgical resection, even for recurrent lesions in patients above 65, stems from evolving evidence that indicates not just feasibility but potential benefits in carefully selected cases.

Firstly, the assertion that advanced age (≥65 years) should not be deemed an independent risk factor for poor outcomes is substantiated by findings from various studies. Age did not significantly impact survival outcomes in elderly patients with brain metastases treated with stereotactic radiosurgery, suggesting that older patients could derive comparable benefits from aggressive treatments [6].

The role of surgical resection, especially in the context of recurrent brain metastases, gains support from studies, which highlighted that complete surgical resection followed by radiotherapy improved local control and functional independence in patients, including those of advanced age. Such findings underscore the therapeutic potential of resection in managing not just initial but recurrent metastatic events, advocating for a strategy that integrates surgical intervention with subsequent adjuvant therapies to optimize outcomes [7].

Resecting brain metastases in patients older than 65 has been shown to improve functional status and facilitate further treatment, diminishing the impact of age as a prognostic factor after receiving combination therapy [8]. The role of resection in recurrent brain metastases has evolved with advancements in oncology and neurosurgery, emphasizing a multidirectional approach toward managing brain metastases. Studies have identified several aspects of resection’s role in this context. The literature demonstrates that repeated surgeries for individual lesions in patients with later recurrence times may confer a survival benefit [9]. Furthermore, achieving maximal tumor resection in the recurrence setting has been linked to improved survival outcomes, and maximal cytoreduction in older patients is associated with prolonged survival [10,11]. Advanced age correlates with higher complication rates post-surgery owing to age-associated comorbidities [12]. However, advancements in surgical techniques and perioperative care have led to a reevaluation of this risk. Patients who underwent resection of a single brain metastasis followed by radiotherapy had significantly improved survival and quality of life, suggesting that the benefits of surgery could outweigh the risks, even in older populations when patient selection is done judiciously [13]. While surgical treatment for recurrent lesions may enhance clinical status and survival, the importance of meticulous patient selection cannot be overstated [14].

Notably, the literature scarcely represents the role of repeated surgical treatment within an elderly patient cohort. Hence, this investigation seeks to analyze the impact of surgical interventions on the survival and clinical characteristics of patients older than 65 years with single brain metastases who have undergone surgical resection for recurrent lesions. The emphasis on meticulous patient selection reflects a growing consensus on the need for personalized care models. As advanced age is associated with a spectrum of health states, comprehensive preoperative assessments, including functional status, neurological deficits, and systemic disease control, become paramount in identifying candidates who may benefit most from repeated resection of brain metastases.

## 2. Materials and Methods

### 2.1. Patient Characteristics

This study focused on analyzing patient characteristics among individuals diagnosed with brain metastases who were referred to the neurosurgical department of the Technical University of Munich from December 2007 to December 2022. The inclusion criteria for primary surgery were limited to patients presenting with synchronous symptomatic brain metastases. Eligibility for re-resection was determined based on recurrence observed in follow-up imaging or accompanying neurological deterioration. The decision to proceed with re-resection was made in collaboration with the patients and their families, considering their preferences.

A cohort of 219 patients was identified, of which 95 underwent surgical treatment one or more times. Subsequently, a subset of 83 patients, aged 65 years or older, was delineated for detailed examination. The patients’ medical records were comprehensively evaluated, encompassing a variety of parameters such as age at diagnosis, gender, tumor location, the total number of brain metastases (BMs), dates of surgical interventions, pre- and postoperative Karnofsky performance status (KPS) scores, tumor burden before and after surgery, and dates of death or last follow-up. Moreover, information pertaining to adjuvant systemic therapies and radiotherapy received by the patients was meticulously gathered and analyzed. In the current study, we document the re-resection of lesions that had previously undergone surgical intervention. All patients underwent or commenced a comprehensive course of radiotherapy.

### 2.2. Ethical Statement

This research adhered to the stringent ethical norms established by the Declaration of Helsinki and its subsequent amendments, receiving approval from the local ethics committee (reference number 5626:12). The ethics committee dispensed with the necessity of obtaining written informed consent from the participants.

### 2.3. Surgery

The surgical strategy was designed to maximize tumor resection while preserving the functionality of eloquent brain regions. Intraoperative neuronavigation was consistently employed as the standard practice. Additionally, when indicated, other advanced techniques, such as neuromonitoring and fiber tracking, were used to enhance surgical outcomes. The decision-making process regarding surgical intervention was spearheaded by a multidisciplinary neuro-oncology board, accounting for several critical factors, including symptomatic lesions, mass effects, intratumoral hemorrhage, diagnostic ambiguities, and the management of larger posterior fossa tumors, which harbor a risk of inducing herniation and hydrocephalus.

### 2.4. Volumetric Analysis

The assessment of the surgical efficacy in terms of tumor removal was facilitated through volumetric measurements conducted on early postoperative magnetic resonance imaging (MRI) scans (within 72 h post-surgery), using T1-weighted sequences enhanced with gadolinium contrast. These measurements determined the volume of residual tumor tissue. Contrast-enhancing tumor components were segmented manually, employing the Origin^®^ software (version 3.1, Brainlab AG, Munich, Germany), with analyses performed by an experienced neuroradiologist and neurosurgeon (the senior author).

### 2.5. Statistics

Statistical data analysis was conducted with SPSS (version 29.0.1.0; IBM, Chicago, IL, USA), along with GraphPad Prism (version 8.3.1; La Jolla, CA, USA). Statistical significance was adjudged at a probability of error threshold of less than 0.05. Descriptive statistics were generated to present demographic variables, encompassing either means with standard deviations or medians accompanied by interquartile ranges, as appropriate. Furthermore, survival analysis was conducted employing Kaplan–Meier estimates to facilitate univariate analysis, complemented by the Cox regression proportional hazards model to execute multivariate analysis. DATAtab eU (Graz, Austria) was used for graphical representation.

## 3. Results

We initially sought to ascertain whether older patients, categorized as aged 65 years and above, experienced a distinct benefit from undergoing re-surgical intervention when juxtaposed with patients younger than 65 years. The evaluation of overall survival following the reoperation was conducted among 36 patients aged 65 years and above and 59 patients under the age of 65 years; the clinical characteristics of all patients with recurrent metastases were reported previously [15]. Of these, 59 patients were younger than 65 years old, consisting of 27 females (45.8%) and 32 males (54.2%), and 36 were aged 65 years and above, comprising 16 females (44.4%) and 20 males (55.6%). The median KPS score both pre- and postoperatively was 70 (IQR: 70–80), improving to 80 (IQR: 70–80) in the older patient cohort. In the younger patient subgroup, 13 (22%) had one metastatic lesion, 14 (24%) had two lesions, and 32 (54%) had three or more lesions. In comparison, among the older patients, 11 (30.6%) had one metastasis, 6 (16.7%) had two lesions, and 19 (52.7%) had three or more lesions. Additionally, systemic progression at the time of initial diagnosis was identified in 26 younger patients (44.1%). Conversely, 16 older patients (44.4%) presented with systemic disease progression.

**Figure 1:**
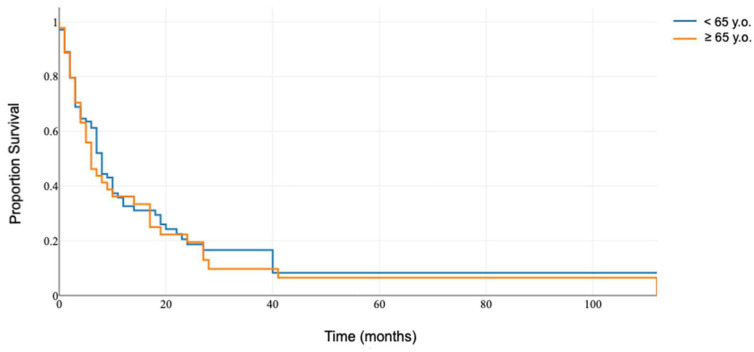
Overall survival of patients after re-resection for recurrent brain metastases.

The investigation revealed an absence of significant differences in overall survival post-reoperation between the two cohorts, with median survival times reported at 6 months (IQR: 4–10 months) for the older patient group and 8 months (IQR: 7–9 months) for the younger patient cohort, yielding a *p*-value of 0.619 (Figure 1).

**Figure 2:**
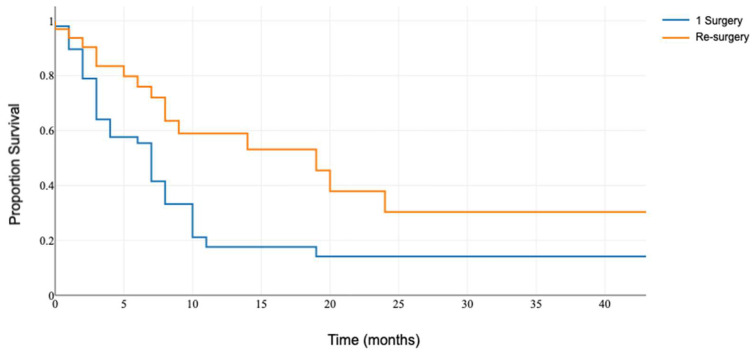
Overall survival of patients aged 65 and older who did and did not undergo re-resection.

After the initial examination, we compared outcomes within the cohort of patients aged 65 and older, specifically evaluating whether undergoing repeated surgical interventions conferred any survival advantage over those who did not receive surgical therapy upon brain metastasis recurrence. The analysis revealed that older patients did indeed benefit from undergoing reoperation following recurrence, highlighting the significance of surgical intervention in enhancing survival prospects for this patient demographic. The median OS was 19 months in older patients who underwent repeated surgery (IQR: 8–24) and 7 months for the patients who did not receive surgical treatment for recurrent lesions (IQR: 4–7): *p* = 0.011 (Figure 2).

We then asked if the functional status could be improved with this treatment strategy. The Karnofsky performance status was remarkably high in the cohort of patients who underwent reoperations for brain metastasis recurrence. All patients had preoperative Karnofsky performance scores of >70, which did not deteriorate after surgery (87.02 ± 5.76 vs. 85 ± 6.85, *p* = 0.055).

**Figure 3:**
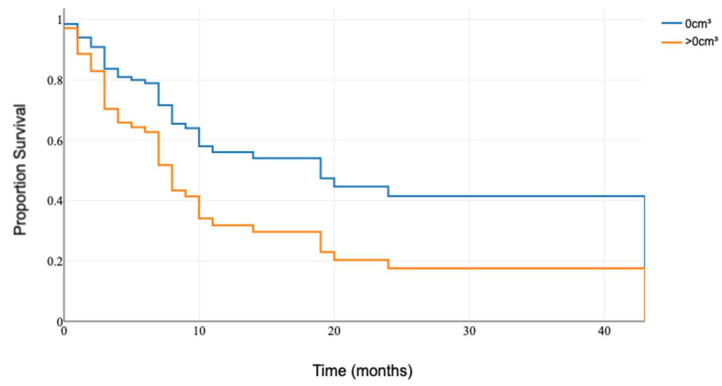
Kaplan–Meier curve comparing patients over 65 with complete and incomplete cytoreduction.

The Cox proportional hazards model analyzed the possible factors affecting the survival of patients. In the univariate analysis, complete cytoreduction was identified as a favorable prognostic factor (Table 1; Figure 3). The tumor volume, the number of metastases, extracranial disease progression, adjuvant radiation, and systemic therapy did not affect survival in this cohort.

## 4. Discussion

The primary objective of this investigation was to scrutinize the outcomes and implications of surgical resection for recurrent brain metastases in the elderly, gauging both survival benefits and quality-of-life improvements post-intervention. Notably, our findings contribute to the nuanced discourse surrounding the surgical management of brain metastases in older patients, a growing demographic due to advancements in systemic cancer therapies and increasing life expectancies.

The surgical management of primary brain metastases in elderly patients continues to be a subject of debate. The principal outcome underscored that elderly patients undergoing repeated surgical resections for brain metastases exhibited prolonged therapy durations and enhanced overall survival, closely echoing recent studies [16,17]. This observation highlights the potential for surgical intervention to significantly impact the treatment paradigm for this patient group.

The surgical resection of brain metastases in older patients embodies a nuanced challenge: balancing symptomatic relief and quality of life enhancements against the inherent surgical risks in this demographic. Amidst an aging global population and the projected uptick in brain metastasis incidences due to improved cancer survival rates, our discussion ventures beyond mere surgical outcomes. It delves into the critical aspects of patient selection, integrating multimodal therapies, and the ethical considerations that orbit the decision-making process regarding surgical interventions in the elderly [18,19,20].

Our study observed no significant differences in survival post-repeated surgery between older and younger cohorts, potentially attributable to the selection of healthier older individuals for repetitive treatments. This underscores the imperative to extend patient selection criteria beyond mere chronological age to embrace physiological age, comorbidities, and functional status. Endorsing comprehensive geriatric assessments can more accurately gauge an older patient’s suitability for surgery, thereby identifying those poised to benefit from surgical resection irrespective of their advanced age [8].

The literature suggests that older patients can achieve significant symptomatic relief from surgical resection but may face higher risks of postoperative complications relative to younger patients. Increased complication rates include surgical site infections, hemorrhagic events, and longer recovery times, potentially affecting morbidity and mortality. However, advances in surgical techniques and technologies, such as minimally invasive approaches and intraoperative imaging, continue to reduce these risks, enabling safer resections even in challenging cases [5,18]. All patients were highly selected for repeated surgery, as per their KPS scores. We also did not observe significant KPS deterioration after surgical resection.

Surgery’s role in treating brain metastases in older adults must be contextualized within a multimodal treatment framework. A significant development in this space is the integration of postoperative stereotactic radiosurgery (SRS), offering a favorable balance between local control and preserving cognitive function and avoiding the neurocognitive sequelae commonly associated with whole-brain radiation therapy (WBRT). Furthermore, the advent of targeted therapies and immunotherapy has revolutionized treatment paradigms, providing opportunities for personalized care strategies that can extend survival and enhance the quality of life of older patients [21,22]. When the role of adjuvant treatment after re-resection was analyzed, no difference was observed in terms of survival.

Maximal resection of brain metastatic lesions is a favorable prognostic factor even in older patients [23,24,25]. The difference in survival rates can be seen after reputed surgeries for metastatic recurrence, highlighting the importance of tumor reduction in elderly patients even during recurrence.

The retrospective design constitutes a limitation of our study, alongside the preoperative KPS scores, which suggest that the involved older patients selected for repeat treatments were clinically stable and likely in a better condition than their counterparts. This emphasizes the essence of customizing decision-making to individual patient circumstances, affirming the importance of adopting a granular, patient-centric approach to the surgical management of brain metastases in the elderly.

## 5. Conclusions

The evolving landscape of neuro-oncology offers promising avenues for enhancing the management of recurrent brain metastases in the elderly. The synthesis of surgical innovations, multidisciplinary care approaches, and a deeper understanding of age-related considerations presents a compelling case for the re-evaluation of surgical resection’s role in this context. Surgical resection of recurrent brain metastases in older patients is a complex process requiring a holistic and individualized approach. We believe that repeated surgery for maximal tumor cytoreduction is a feasible option for elderly patients, leading to prolonged survival. As surgical techniques and adjuvant therapies continue to advance, the potential to offer meaningful surgical interventions to older patients with brain metastases increases. Ongoing research dedicated to this age group is essential for optimizing outcomes, minimizing risks, and ensuring that treatment decisions align with the broader spectrum of patient care preferences and ethical considerations.

## Figures and Tables

**Table 1:** Cox regression analysis of risk factors for overall survival in patients who underwent repeated surgical resection.

Name	Coefficients	Lower 95% CI	Upper 95% CI	Std. Error	z	*p*	Exp(B)	Lower 95% CI	Upper 95% CI
Tumor volume	0.03	0.01	0.05	0.01	2.58	0.01	1.03	1.01	1.06
KPS (≥80 vs. 70)	−0.19	−2.21	1.82	1.03	0.19	0.851	0.82	0.11	6.17
No. of lesions (≤3 vs. >3)	1.55	0.27	2.83	0.65	2.37	0.018	4.72	1.31	16.97
Systemic therapy	0.14	−0.79	1.06	0.47	0.29	0.773	1.15	0.45	2.9
Radiation	−0.1	−0.74	0.53	0.32	0.32	0.749	0.9	0.48	1.7
Combination treatment	−0.21	−0.77	0.36	0.29	0.72	0.472	0.81	0.46	1.43
**Total resection**	**0.68**	**0.01**	**1.35**	**0.34**	**1.98**	**0.048**	**1.98**	**1.01**	**3.87**

## Data Availability

The original contributions presented in the study are included in the article, further inquiries can be directed to the corresponding author.
